# Caries Level in 3-Year-Olds in Germany: National Caries Trends and Gaps in Primary Dental Care

**DOI:** 10.3390/children11121426

**Published:** 2024-11-26

**Authors:** Ruth M. Santamaría, Christian H. Splieth, Roger Basner, Elisabeth Schankath, Julian Schmoeckel

**Affiliations:** Pediatric Dentistry Department, University Medicine of Greifswald, 17475 Greifswald, Germany; ruth.santamaria@uni-greifswald.de (R.M.S.); splieth@uni-greifswald.de (C.H.S.);

**Keywords:** caries prevalence, caries index, 3-year-olds, Germany

## Abstract

Background: Nationally representative long-term data on caries in the primary dentition are rare but essential for determining the need for prevention and treatment. This research assessed the prevalence and trends of dental caries in 3-year-old children across Germany, with national data analyzed and compared with the corresponding data for 6–7-year-olds. Methods: Data were extracted from the most recent German National Oral Health Survey in 2016. Children aged 3 years were examined by calibrated dentists in 10 German regions using the WHO criteria for d_3–4_mft, including assessment of initial carious lesions d_1–2_mft. In addition, the Significant Caries Index (SiC), the Care Index (CI) and the Specific Affected Caries Index (SaC) were considered to identify provision of care and risk groups. Results: In a total of 95,127 3-year-old preschool children, caries prevalence was 13.7% with a mean d_3–4_mft of 0.48. Including initial carious lesions, prevalence increased to 18.7% (mean 0.67 d_1–4_mft). Dependent on the German region, d_3–4_mft values varied noticeably from 0.38 (Schleswig-Holstein) to 0.58 (Saxony-Anhalt and Berlin). Comparing data from 3-year-olds to 6–7-year-olds, the d_3–4_mft value for 6–7-year-olds (1.73) was more than three times higher than that for 3-year-olds (0.48). The SiC value was 1.47 for 3-year-olds and 4.88 for 6–7-year-olds, while the SaC values were 3.57 and 3.97, respectively. The Care Index was low for both groups (26.1% and 57.5%, respectively). Conclusions: Germany exhibited a high level of dental caries in the primary dentition for 3 (13.7%) to 6–7-year-olds (44%) children. This large cross-sectional study revealed considerable room for improvement in the early caries prevention and treatment within the well-equipped German dental health infrastructure.

## 1. Introduction

Dental caries is a manageable chronic disease of dental hard tissues that can be diagnosed early and minimally invasively treated [1,2], yet it affects a vast number of individuals globally [3,4]. An estimated 2.4 billion people experience caries in permanent dentition, and 486 million children have caries in the primary dentition, with significant regional variation [5,6,7]. European countries show lower prevalence rates, while African countries have higher rates [8].

Despite improvements in caries levels among children with permanent dentition, such as the 80% reduction in DMFT values among 12-year-olds in Germany from 1994/1995 to 2016, the distribution of caries remains polarized, heavily affecting socio-economically and geographically underprivileged groups, with children from lower socio-economic backgrounds experiencing about 60% higher DMFT levels than their higher socio-economic counterparts [9,10].

On the other hand, for primary dentition worldwide, to date, only a few national studies [11,12,13] and several regional studies with limited sample sizes have been documented [11,14,15,16,17]. These studies often utilized specialized, unrepresentative samples such as dental clinic attenders, and exhibited wide age ranges in the study population, often lacking representation of very young children in most sample groups [14,18]. Even in Germany, where longitudinal data from the sequential cross-sectional German National Oral Health Surveys (GNOHSs) in children have been regularly collected since 1994/95 for 6–7-year-olds [10,19] and for 12-year-olds [9,10] there is a notable absence of representative data at the national level for preschoolers. Only very few studies from localized regions in Germany have focused on assessing the caries prevalence of this very young group [20,21].

In addition, to accurately assess prevention and treatment needs, it is essential to look beyond mere prevalence levels and consider the entire caries experience and its distribution within a population. A truly comprehensive understanding requires a detailed description and analysis of not only the mean dmft (decayed, missing, and filled teeth) index and its individual components but also the presence of initial carious lesions. This is particularly crucial for countries with lower caries levels across various reference groups. Additionally, it is imperative to evaluate restorative care and incorporate indices displaying the polarized distribution (e.g., Significant Caries Index [22], Specific Affected Caries Index [23]) and highlighting high-risk groups within a population accurately. This comprehensive approach enables a more holistic assessment of current trends in dental health and ensures that preventive as well as treatment efforts are appropriately targeted towards those who need these most.

The main objectives of this report are:

- To provide an analysis of nationally representative data on the prevalence of enamel and dentinal caries in 3-year-old children attending mainstream nurseries/kindergartens across Germany.
- To compare these findings with national data for 6–7-year-olds to assess caries trends in the primary dentition.
- To identify ongoing challenges related to caries burden and treatment needs in the primary dentition.

## 2. Materials and Methods

### 2.1. Ethical Aspects

Ethical approval for this study was granted by the Research Ethics Committee of the University of Greifswald (Reg.-Nr.: BB48/10a). The study design and implementation adhered to the fundamental principles set forth in the Declaration of Helsinki (1964) and its subsequent revisions.

The data used for this report were based on published, nationally available free-access data: https://daj.de/wp-content/uploads/2024/02/Epi_final_BB0103_final_Druckvorbereitung.pdf, accessed on 6 September 2024 from the latest German National Oral Health Surveys (GNOHS) [10].

### 2.2. Study Framework and Population

The German Association for Dental Prevention in Children and Adolescents (DAJ), representing 17 regional associations (LAGs), oversees the execution of caries preventive measures in schools and kindergartens, alongside conducting epidemiological surveys. These actions are predominantly led by public dentists and preventive dental assistants mandated to conduct regular surveys to gauge program effectiveness. However, in selected federal states, specialized teams or private dentists may undertake these responsibilities. This structure has enabled the execution of five consecutive German National Oral Health Surveys (GNOHSs) spanning from 1994/95 to 2009, as well as the latest survey in 2016 [10]. The inclusion of 3-year-old preschool children in the survey for the first time was a notable development in the latest GNOHS. The data for the present study were derived from examinations in nurseries/kindergartens conducted on 3-year-olds across various German states.

For the epidemiological examination, the LAGs could choose either to submit their regular community data attempting a full survey (6/10 regions) or data from a representative sample (2/10 regions). The latter samples were drawn through a randomized cluster selection process by the DAJ in cooperation with the GESIS Leibniz Institute for the Social Sciences (Mannheim/Germany) based on data from the National Health Reporting System (Gesundheitsberichterstattung des Bundes—GBE) from the lists of all nurseries/kindergartens in 2014. In cases where complete lists of nurseries/kindergartens were unavailable (2 regions), the examination of all possible 3-year-old preschoolers took place in the available nurseries/kindergartens.

### 2.3. Sampling

Several LAGs that chose a sampling method (where a subset of the population was selected to represent the whole) or a complete survey approach (where data were collected from the whole population) slightly missed the target sample size needed to achieve a confidence level of at least 70% for representing the study population, as lower response proportions may not necessarily cause higher bias [24]. Still, only very few regions (Lower Saxony and Schleswig-Holstein) achieved low proportions. Nonetheless, a notably large number of children (several thousand) underwent examination in each region (Table 1), resulting in an overall mean dmft value calculated across regions. The impact of imperfect fulfillment of predetermined criteria by these selected LAGs on the overall value portraying the mean caries experience for Germany is considered to be minimal [10] as a very detailed statistical adjustment of caries levels in 12-year-olds for each region revealed also almost negligible effects on mean national DMFT [25]. In addition, all caries values for 3-year-olds were in a relatively narrow range. By performing a ranking of the federal states, their ranking corresponds to the ranking in the groups of 6- to 7- and 12-year-olds from the same GNOHS, which makes the data extremely plausible [10].

### 2.4. Clinical Examination and Calibration of Examiners

Training and calibration of examiners was carried out using a methodology previously published [9,10,19], which followed the consensus guidelines for “Standardized Reporting of Oral Health Data” in German community public services at that time [26]. In summary, this involved visual examination using the WHO caries severity index (d_3–4_mft) for primary teeth (carious: dt, missing: mt, and filled teeth: ft). In addition, the presence of initial carious lesions (d_1–2_), referring to non-cavitated lesions limited to the enamel, was counted in.

Examiners could be either dentists routinely working as a public health dentist performing intra-oral examinations on a regular basis in schools and kindergartens, or private dentists involved in public health caries prevention, or study dentists specifically assigned to examine for this study. All had to successfully pass the online calibration described below before starting to collect data for this study, irrespective of their background. This was assured by the regional project coordinators in the LAGs.

Oral examinations of the participants were conducted nationwide from May 2015 to August 2016. Examiners (n = 482) underwent calibration using a dedicated online platform tailored for this task. The e-learning system included theoretical learning, practical exercises utilizing clinical images, and a calibration module using a randomized sequence of 55 images. For all examiners, the inter-examiner weighted kappa rank values were computed [27,28]. All inter-examiner weighted Kappa rank values exceeded 0.65, indicating a substantial level of agreement. Specifically, 212 examiners (44%) achieved substantial agreement (K = 0.65–0.80), while the remaining 270 (56%) attained (nearly) perfect agreement (K = 0.81–1.00).

### 2.5. Statistical Analyses and Data Recording

The data were predominantly captured digitally utilizing either the dedicated software employed by public health services or, otherwise, in a previously standardized Microsoft Excel^®^ datasheet. In cases where digital recording was not feasible, a printed datasheet was utilized for manual recording. The recording and subsequent data transfer to the regional LAG for analysis were conducted anonymously, capturing only gender, date of birth, examination date, and d_1–4_mft values per child.

A comprehensive quality check was conducted to ensure accurate data transfer from the examiners to the regional LAGs and ultimately to the analysis team for final statistical processing. Since some results relied on secondary data from the LAGs, an initial raw dataset was securely stored and subjected to a plausibility check. This process provided a standardized evaluation dataset for each state, with all quality assurance steps thoroughly documented. Additionally, quality management included filtering age and grade data to standardize datasets, ensuring that only sampled institutions were included and any extraneous data were removed and documented. For this study, data documentation methods were flexible yet pre-standardized, allowing for the use of pre-designed paper forms, Excel^®^, or digital recording through various software platforms. Digital recording, in particular, minimized errors, streamlined data transmission, and enabled immediate local analysis. Centralized data collection within the LAGs allowed for preliminary checks, further enhancing data quality.

All caries data related to 3-year-olds were included for analysis. The caries data were subjected to descriptive analysis, examining prevalence, mean d_3–4_mft, and the individual components (d, m, f, and initial carious lesions [d_1–2_]). Additionally, we calculated:

- The Care Index (CI) as the number of restored and extracted teeth due to caries as a fraction of the total number of d_3–4_mft.
- The Significant Caries Index (SiC) as the mean d_3–4_mft value of the one-third of children with the highest caries levels [22].
- The Specific Affected Caries Index (SaC) as the mean d_3–4_mft among individuals with d_3–4_mft > 0 to identify risk groups in populations with low caries prevalence [23]. To calculate the SaC, we employed the following method: mean d_3–4_mft in the group of individuals with d_3–4_mft > 0. Alternatively, if caries prevalence and mean d_3–4_mft were known, the formula used was as follows: 100%/caries prevalence in % × mean d_3–4_mft.

For the national data, the mean values of the participating federal states were weighted according to the relative size of the population of 3-year-old children. Data from 6–7-year-olds from the same German regions were used for presentation of caries trends and subjected to analogous descriptive analysis [10,19].

## 3. Results

### 3.1. Sample Distribution

A total of 95,127 three-year-old preschool children (51% boys and 49% girls) were linked to 10 German regions and included in this analysis.

### 3.2. Caries Experience and Treatment Needs in 3-Year-Olds

The weighted mean d_3–4_mft for 3-year-olds in Germany was 0.48 (dt = 0.36; mt = 0.04; ft = 0.08). With inclusion of initial carious lesions, the score for caries experience increased to d_1–4_mft = 0.67 (d_1–2_ = 0.19). With respect to the German region, the mean d_3–4_mft varied within a narrow band, presenting a range from 0.38 in Schleswig-Holstein to 0.58 in Saxony-Anhalt and Berlin (Table 2).

The proportion of 3-year-olds who were “caries free” (no cavitated carious lesions, thus d_3–4_mft = 0) was 86.3%. The proportion of ‘caries-free’ children was reduced to 81.3% when initial carious lesions were included in the index. The percentage of 3-year-olds with manifest caries (d_3–4_mft > 0) varied between 10.5% (Schleswig-Holstein) and 16.6% (Saxony-Anhalt) in the different German regions (Table 2), rising to 14.4% and 24.6% when initial lesions were included.

Only 2.3% of the preschoolers’ dentitions were treated, leaving 11.4% of them with a need of some restorative/surgical treatment. Children with caries experience (d_3–4_mft > 0) had a mean d_3–4_mft = 3.55 (SaC).

At the tooth level, 73.9% of primary teeth with dentine caries (d_3–4_) remained untreated, 8.7% were treated by extraction (m), and 17.3% had an intact filling (f). The Care Index was 26.1%.

### 3.3. Evaluation of Caries Levels in Primary Teeth of 3- and 6–7-Year-Old Children

Reported caries data from Germany for 3- and 6–7-year-olds [10,19] were compared (Table 3). The d_3–4_mft values for 6–7-year-olds (1.73) were more than three times higher than those for 3-year-olds (0.48). When initial carious lesions were considered, the difference increased, to 2.11 vs. 0.67, respectively. As the caries prevalence was substantially higher in 6–7-year-olds than in 3-year-olds, the proportion of children requiring treatment (d_3–4_ > 0) also rose, from 11.4% to 26.9%. Conversely, the number of caries free children (d_3–4_mft = 0) decreased by about 34.7%.

Analyzing the components of the d_3_mft values, the d_3–4_-component made up 75% of the total index for 3-year-olds, compared to 42% for 6–7-year-olds. At the regional level, Schleswig-Holstein and North Rhine consistently showed the highest proportions of caries-free children among 3-year-olds (89.5% and 61.5%, respectively) and 6–7-year-olds (88.8% and 61.4%, respectively). These German states also had the lowest numbers of children needing dental care (Table 3).

**Figure 1:**
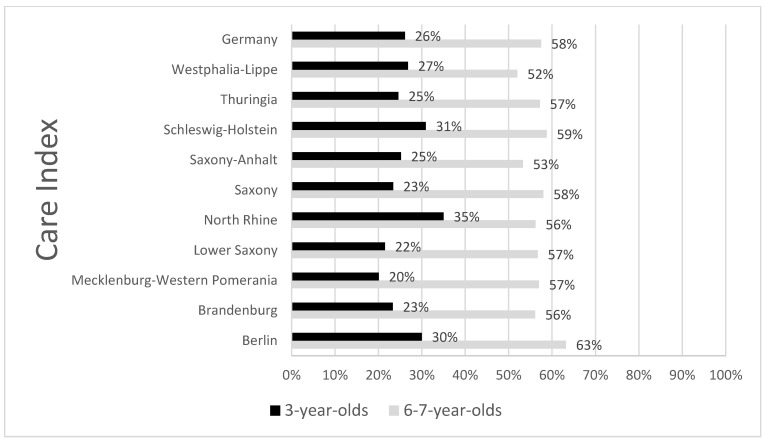
Care index for primary dentition in 3- and 6–7-year-olds in Germany. Data adapted from [10].

The SiC d_3–4_mft value was 1.47 for the 3-year-olds and 4.88 for the 6–7-year-olds and the Care Index was 26.1% and 57.5%, respectively (shown in Figure 1). Comparisons of regional data for SiC showed homogeneous values for both age groups (Table 3). The SaC values of the total samples were 3.57 and 3.97, respectively (Table 3).

Comparing caries trends separately for 3-, 6-, and 7-year-olds, there was a clear tendency for caries levels to increase markedly with age, as shown in Table 4. In general, the prevalence of caries increased from 13.7 to 43.6% (>300%) and the mean d_3–4_mft from 0.48 to 2.08 in children between the ages of 3 and 7 years.

## 4. Discussion

This report presents an analysis of the caries experience of 3-year-olds from the latest GNOHS [10] and a comparison of this age group with data, also national, for the reference group of 6–7-year-olds. As 3-year-olds were included in the GNOHS for the first time, key data for regional and national dental indicators could be obtained. These findings contribute to better insights on caries experience and the dental care provided to, or still needed by, young children in Germany.

The main outcomes indicate a relatively high caries prevalence of 13.7% in 3-year-old preschoolers from a high-income country (Germany), with a mean d_3–4_mft of 0.48, which increased greatly in the following four years to 2.08 (7-year-olds). Moreover, the Care Index was irresponsibly low (26.1% for 3-year-olds), increasing but remaining unsatisfactorily low even for 6–7-year-olds (57.5%), even though dental care for primary teeth is fully covered by the German public health insurance system [29]. Additionally, the polarized distribution and the low Care Index especially in 3-year-olds reveals the challenge of a relevant fraction of all children (~5%) being potential candidates to receive dental rehabilitation under general anesthesia with its risks for the children and burden (cost and practical efforts) for the health system and the providers. This requires action for prevention and the need for possibly better educated dentists as well as more pedodontics specialists, which will be discussed in more detail below.

When comparing data from 3-year-olds and 6–7-year-olds, several age-related factors may have influenced the study results. The differences in the development and progression of caries according to age play an important role. In our study, the younger cohort (3-year-olds) showed a lower prevalence of caries and a higher proportion of initial lesions, probably due to their limited age-related exposure to cariogenic factors. Despite the lower overall caries prevalence in the younger group, the Care Index indicated that they received less dental care than the 6–7-year-olds, highlighting age-related differences in treatment needs and access to care.

Worldwide, caries levels in primary dentition continue to show alarming figures. According to a recent systematic review on the global prevalence of early childhood caries (ECC) that includes 64 reports from 67 countries, spanning 1992 to 2019, the global pooled prevalence of ECC was 48% (95% CI: 43–53). A significant variation could be observed by continent: Africa (30%), Americas (48%), Asia (52%), Europe (43%), and Oceania (82%). These results highlight that ECC is a highly relevant global health concern with nearly half of preschool children, with considerable variability in prevalence across regions and limited dental care [18]. In Germany, the latest GNOHS assessed caries prevalence and caries trends (1994 to 2016) in 6- to 7-year-olds [10,19]. Caries prevalence in this age group decreased by around 32% from 1994 to 2016, with the mean caries experience decreasing from 2.89 to 1.73 d_3–4_mft. However, a high SiC of 4.84 was observed, highlighting the severity of caries lesions in those most affected. In the current analysis, 3-year-olds exhibited a lower SiC of 1.47, which might be misinterpreted as indicating that the disease is not severe in this age group. However, caries prevalence in this age group comprises less than one-third of the population, meaning that many caries-free children (d_3–4_mft = 0) are included in the SiC calculation. In this context, the SaC (3.57) provided a more precise assessment of the actual polarized distribution by measuring the mean d_3–4_mft among children with any caries experience (d_3–4_mft > 0). This approach offers a more accurate representation of caries severity within the affected subgroup, effectively highlighting the true severe extent of the disease with clinical relevance [23].

In Germany, oral health preventive policies are mainly uniformly realized across the country. In spite of significant success in reducing caries levels in permanent teeth, the data underlines only limited improvement in the primary dentition over the past decades. To analyze this issue, it is important to recognize the strong link between caries reduction and fluoride use [30,31], with Germany historically using a low concentration of fluoride (500 ppm Fl) in children’s toothpaste at the time of the dental examination for this research. Additionally, caries prevention programs for children in nurseries and kindergartens have been significantly less developed compared to those for school-aged children. Fortunately, since the publication of the latest caries prevalence data in Germany for different children age groups [10,19,32], new national caries control policies have been implemented. Since 2018, Germany has adopted the European recommendations [31] to use higher fluoride concentrations in children’s toothpaste (>1000 ppm Fl). Furthermore, from mid-2019, early oral health prevention strategies for primary teeth have been expanded to insurance-paid dental check-ups and caries risk evaluation starting with the eruption of the first primary tooth (FU1a-c), fluoride varnish applications (FLA) evaluated to be effective, and practical tooth-brushing training with caregivers (FUPr) [29]. In addition, pediatricians should use the revised “pediatric examination booklet” (German: Kinder-Untersuchungsheft), which was adjusted in 2016, to refer young children to early dental examinations during the mandatory pediatric examinations (U5-U7) and thus promote ECC prevention [32]. A pilot project in Jena has already demonstrated the effectiveness of early caries prevention with the introduced toolkit for the population of that city [33]. The above-mentioned measures are in line with the ‘International Association for Pediatric Dentistry (IAPD) Bangkok Declaration’ [34] on ECC. To prevent ECC, parents, dentists, doctors, and many other stakeholders should be sensitized to ECC, and preventive counselling should be provided by a healthcare professional within the first year of life if possible. Ideally a referral should be made to a dentist for comprehensive care. All these new measures are now contained in the German National Health Catalogue and are financially covered [29]. Given the existing favorable evidence supporting these strategies [35,36], the efficacy of early prevention in caries control in children will become increasingly visible over time, provided they are implemented effectively. It is essential to highlight that strategies to control caries depend heavily on parental adherence to tooth-brushing guidelines and regular dental visits for their children. This can be particularly challenging for high-risk groups. The impact of these new national strategies should be further evaluated and examined in future epidemiological studies. The future will show to what extent caries can be avoided in small children as, e.g., the Alliance for Cavity Free Future (ACFF) aims at for all children born after 2030 [37], similar to the manual of the WHO “Ending childhood dental caries” [38].

Current caries data for 3-year-olds show that only about 25% of carious teeth have been treated, either by filling or extraction. For 6- to 7-year-olds, this figure improves but remains unsatisfactorily low, with just over half of the carious teeth treated nationally. Remarkably, the Care Index at the national level remains unsatisfactory, in a country where the public health insurance covers almost all preventive, restorative (e.g., preformed metal crowns, pulp treatment, as well as fillings and extractions), and rehabilitative (e.g., removable space maintainers, early orthodontic treatment) care provided to children, regardless of whether the care is provided chairside or under general anesthesia. Therefore, there is still a clear need for improvement, not only in the prevention of caries, but also in the treatment of carious lesions in the primary dentition. Another possible major reason for the inadequate Care Index in this cross-sectional study may also be the high failure rate of the provided restorative care in Germany [39]. A recent long-term retrospective study of German health data over a comprehensive seven-year period shows that fillings in primary molars are very common. However, depending on the size of the filling, they only have a success rate of 46.2–52.6%, with multi-surface fillings failing more often than single-surface fillings.

There are no recent national data on caries in the primary dentition other than those reported here, probably due to the COVID-19 pandemic, but regional data are available. For the federal state of Brandenburg, representative cross-sectional data for 3- and 6-year-olds are available, showing annual improvements from 2016 to 2023 in the proportion of caries free (3 y/o = 86.5% to 91.7%; 6 y/o = 56.7% to 65%) and a decrease in the mean d_3–4_mft (3 y/o = 0.48 to 0.29; 6 y/o = 1.68 to 1.32) [40]. Although only regional data are currently available, it is clear and plausible that similar trends of slight improvements in dental health have occurred in the other regions [41].

The observed insufficient provision of dental care (shown in Figure 1 and Table 3) and the high reported failure rate for restorative dental care [36] most likely reflect limitations in the ability to treat young children at country level. This may also be influenced by the limited undergraduate training of dentists. For instance, the use of long-lasting restorative materials for primary molars, such as preformed metal crowns (PMCs) in daily practice, is still very limited, despite the vast evidence proving that they are much more successful than multi-surface fillings [42,43,44,45,46]. This is particularly important when considering the polarized caries distribution in line with caries risk and caries activity. Here, PMCs clearly outperform fillings because of the persistent caries activity/secondary caries.

A major asset of this study is the integration of data on caries at the threshold of initial non-cavitated lesions, which allows earlier identification of caries risk and prioritization of preventive and minimally invasive treatment approaches. Nonetheless, the PUFA index [47,48] could be added as relevant information regarding clinical consequences of untreated dental caries to traditional caries indices (like dmft/DMFT) for epidemiologists and health policies. This may also correlate more strongly to the children’s oral-health-related quality of life, which often is assessed differently by patients/parents and the dental care providers [49,50].

These data from a national epidemiological survey provide valuable insights for more efficient allocation of financial resources [51], focusing on preventive measures rather than costly interventions required for treating cavitated/advanced lesions. Hence, health systems can invest more effectively in sustainable oral health strategies. On the other hand, caries activity assessment was not included in this study. Although this would have been valuable, particularly for understanding true treatment needs, evidence from studies assessing caries activity using the ICDAS criteria suggests that including caries activity in epidemiological surveys could help to refine treatment needs with limited effect on caries prevalence estimates [52]. Another limitation of this epidemiological study is the potential variability of the data due to possible differences between regions. Differences in diagnostic criteria and health policies could influence the results, potentially affecting consistency [53]. However, rigorous calibration procedures were used in this study to align assessment standards across all regions, thereby increasing the reliability of the data. In addition, the participating examining dentists regularly examine children as part of regular kindergarten/school examinations and not exclusively for this study, which strengthens the consistency and accuracy of the data collected. Furthermore, data from the 6–7-year-old cohort showed comparable results to another epidemiological study in Germany [19], supporting the validity and generalizability of this study’s outcomes.

## 5. Conclusions

The primary dentition of 3–7-year-olds in Germany still exhibits high caries prevalence along with an unsatisfactory low level of care. The representative data of this large cross-sectional national study highlight the need and potential for improvement in ECC prevention and treatment within the country’s robust dental infrastructure. Due to implemented caries preventive measures in recent years, future improvements are to be expected.

## Figures and Tables

**Table 1:** Sample distribution of participating federal states based on relative population size of 3-year-olds in the GNOHS in 2015/2016 [10].

Region	Total Examined 3-y/o	Collection Approach	3-y/o Attending Nurseries ^3^	Examined Proportion of Study Population (%)	All 3-y/o (Destatis) ^4^	Examined Proportion of Total Population (%)
Berlin	16,453	GBE	30,203	54.5%	34,882	47.2%
Brandenburg	14,337	GBE	18,942	75.7%	20,810	68.9%
M-W-P	8241	GBE	11,960	68.9%	13,391	61.5%
Lower Saxony	4870	GBE ^2^	54,997	8.9%	66,819	7.3%
North Rhine ^1^	1849	AS	68,474	2.7%	82,727	2.2%
Saxony	22,479	GBE	33,233	67.6%	36,285	62.0%
Saxony-Anhalt	9415	GBE	16,173	58.2%	17,790	52.9%
Schleswig-Holstein	5530	GBE ^2^	19,136	28.9%	23,848	23.2%
Thuringia	10,523	GBE	16,834	62.5%	18,211	57.8%
Westphalia-Lippe ^1^	1430	AS	59,907	2.4%	72,377	2.0%
**Germany**	**95,127**	**-**	**612,931**	**15.5%**	**713,757**	**13.3%**

M-W-P: Mecklenburg–Western Pomerania; GNOHS: German National Oral Health Survey; 3-y/o (3-year-olds); GBE: German National Health Reporting System (German: Gesundheitsberichterstattung des Bundes); Destatis: German Federal Statistical Office (German: Statistische Bundesamt). ^1^ AS: available samples. These were used in two regions: in North Rhine 53.6% and in Westphalia-Lippe 57.7 % of the samples were examined. ^2^ Lower Saxony and Schleswig-Holstein examined representative samples. ^3^ Data on 3-y/o attending nurseries were recorded in the school year before the examination (Destatis). ^4^ All 3-y/o living in the federal state in the school year of the examination (Destatis).

**Table 2:** Caries experience in the primary dentitions of 3-year-old children attending kindergarten or nurseries in Germany *.

Caries Levels in 3-Year-Olds							Caries Prevalence		GA Risk
Region	d_3–4_mft	d_3–4_t	mt	ft	d_1–2_t	d_1–4_mft	d_1–2_t > 0 (%)	d_3–4_mft > 0 (%)	d_3–4_mft > SaC (≥4) (%)
Berlin	0.58	0.41	0.05	0.13	0.29	0.87	8.4%	16.2%	6.2%
Brandenburg	0.48	0.37	0.03	0.08	0.17	0.65	4.8%	13.5%	5.0%
Mecklenburg–Western Pomerania	0.51	0.41	0.03	0.07	0.18	0.69	5.5%	14.5%	5.3%
Lower Saxony	0.52	0.41	0.04	0.07	0.14	0.66	5%	13.8%	5.5%
North Rhine	0.39	0.26	0.05	0.08	0.18	0.57	3.6%	11.2%	4.1%
Saxony	0.40	0.31	0.03	0.07	0.16	0.56	5.6%	11.6%	4.4%
Saxony-Anhalt	0.58	0.43	0.05	0.10	0.18	0.76	4.2%	16.6%	6.1%
Schleswig-Holstein	0.38	0.26	0.04	0.07	0.16	0.54	3.9%	10.5%	4.2%
Thuringia	0.56	0.42	0.04	0.10	0.25	0.81	4.3%	15.9%	6.1%
Westphalia-Lippe	0.49	0.35	0.06	0.07	0.20	0.69	5.0%	13.2%	5.0%
**Germany**	**0.48**	**0.36**	**0.04**	**0.08**	**0.19**	**0.67**	**5.0%**	**13.7%**	**5.3%**

* Data adapted from [10]. d (decayed); m (missing); f (filled); d_1–2_ (enamel carious lesions); d_3–4_ (dentinal caries). GA risk: Children at high caries risk, as indicated by a high Specific Affected Caries Index (SaC), often require oral rehabilitation under general anesthesia due to the severity of their caries experience. d_1–2_t > 0: Prevalence of children with enamel caries only while having a d_3–4_mft = 0.

**Table 3:** Comparison of caries indices in the primary dentition of 3- and 6- to 7-year-old children in 10 different federal states and Germany as a whole *.

Region	d_3–4_mft		d_1–4_mft		SiC		SaC		Care Index		“Caries Free” Children, d_3–4_mft = 0		Children with Need of Care, d_3–4_ > 0	
	3-y/o	6–7-y/o	3-y/o	6–7-y/o	3-y/o	6–7-y/o	3-y/o	6–7-y/o	3-y/o	6–7-y/o	3-y/o	6–7-y/o	3-y/o	6–7-y/o
Berlin	0.58	2.13	0.87	2.47	1.75	5.67	3.61	4.20	30.0%	63.2%	83.8%	49.4%	13.3%	30.2%
Brandenburg	0.48	1.85	0.65	2.04	1.43	5.03	3.55	3.89	23.3%	56.1%	86.5%	52.4%	11.4%	29.7%
M-W-P	0.51	2.23	0.69	2.53	1.52	5.57	3.50	3.87	20.1%	57%	85.5%	42.6%	12.3%	36.3%
Lower Saxony	0.52	1.78	0.66	2.06	1.56	5.00	3.76	4.11	21.5%	56.7%	86.2%	56.8%	12.0%	28.3%
North Rhine	0.39	1.59	0.57	1.8	1.17	4.60	3.48	4.11	35.0%	56.2%	88.8%	61.4%	9.3%	24.5%
Saxony	0.40	1.75	0.56	2.0	1.21	4.84	3.47	3.82	23.4%	58%	88.4%	54.1%	10.0%	27.9%
Saxony-Anhalt	0.58	2.31	0.76	2.5	1.73	5.88	3.48	4.16	25.2%	53.3%	83.4%	44.4%	13.8%	36%
S-H	0.38	1.47	0.54	1.66	1.15	4.26	3.65	3.82	30.9%	58.8%	89.5%	61.5%	8.2%	22.2%
Thuringia	0.56	2.08	0.81	2.3	1.67	5.45	3.50	3.98	24.6%	57.2%	84.1%	47.7%	13.3%	31.9%
W-L	0.49	1.78	0.69	1.98	1.48	5.02	3.72	4.09	26.8%	52%	86.8%	56.4%	10.6%	29.5%
**Germany**	**0.48**	**1.73 ^1^**	**0.67**	**2.11 ^1^**	**1.47**	**4.84 ^1^**	**3.57**	**3.97 ^1^**	**26.1%**	**57.5% ^1^**	**86.3%**	**56.4% ^1^**	**11.4%**	**26.9% ^1^**

M-W-P: Mecklenburg–Western Pomerania; S-H: Schleswig-Holstein; W-L: Westphalia-Lippe; d_3–4_mft (d = decayed; m = missing; f = filled dentinal caries); d_1–4_ (d_1–2_ = initial carious lesions and d_3–4_mft together); SiC (Significant Caries Index); SaC (Specific Affected Caries Index); CI (Care Index); 3-y/o (3-year-olds); 6–7-y/o (6–7-year-olds). * Data adapted from [10]. ^1^ For the Germany totals for 6–7-year-olds, reported data correspond to the country average.

**Table 4:** Comparison of mean d_3–4_mft trend values for 3-, 6, and 7-year-old children in Germany *.

Region	d_3–4_mft		
	3-Year-Olds	6-Year-Olds	7-Year-Olds
Berlin	0.58	1.99	2.31
Brandenburg	0.48	1.61	2.12
Mecklenburg–Western Pomerania	0.51	2.06	2.33
Lower Saxony	0.52	1.61	1.99
North Rhine	0.39	1.57	1.61
** Saxony	0.40	1.75	1.75
Saxony-Anhalt	0.58	2.15	2.42
Schleswig-Holstein	0.38	1.26	1.72
Thuringia	0.56	1.94	2.22
Westphalia-Lippe	0.49	1.63	1.98
**Germany**	**0.48**	**1.76**	**2.08**

* Data adapted from [10]. ** No differentiated data for 6- and 7-year-olds provided in DAJ report [10].

## Data Availability

The data that support the findings of this study are openly available at https://daj.de/wp-content/uploads/2024/02/Epi_final_BB0103_final_Druckvorbereitung.pdf (accessed on 6 September 2024), reference number [10].
